# Supplementary material for: Chromothripsis during telomere crisis is independent of NHEJ, and consistent with a replicative origin
Source: Genome Res. 2019 May;29(5):737–49. doi: 10.1101/gr.240705.118 (PMC6499312; doi:10.1101/gr.240705.118)
Supplement: Supplemental Material [file supp_gr.240705.118_Supplemental_file_1.zip › contigs/annotated_contigs/DB53/contig.2.DB53_length_772_mean_cov_11.4507772021.docx]

**DB53_length_772_mean_cov_11.4507772021**

```
TTCCACCAAAAACCTATGGAAATAAAAAGTAAAAATAAAATAAATTAAAAAAGAAAAAAATTTTCTTTTAAAGCATCACCTGTGAATCA
 >chr6:68507074-68507469 + E=8e-226
GAGAATCCTCACCAGACACTGAAACTGCTGTCACTTAGGTCTTGGACTTCCCAGCTTCCAAAACTGTGAGAAATATGTTTTTGTTGTTT

ATAGACTCCAGTTTGAAGTGCTTTGTTATAGTAATCAAAATTAACTAAGACAGATGAACACTCCAGGCAGAGGGAGTAGCATTAGCCAC

TATCAAAAGCATGACATGGTACATATGTGTTTTCATATTGTTAGGGCAAAAGTTAAGTGTATTTATAGACTGGTTAGATATTGTCGAAT

TCCCCTCCAGAAAGATTGTGTCATATTACATTCTCAT|GA|ATCATCCGTCGCAACACTAGGAGCTTGAGTTCCAGCAAGGCCCGCAAC
                                      >chr6:68508320-68508699 + E=3e-216
CACAAGCTGAATTGTTCTGGGGCCCTAAATACACTTGAAAGGCAGTCTAGATAGCAAGAACTGCAATTCCTGGACAAGTCCTGATGCTG

TACTAGTCTCAGAGCCAGTGCACTTGGAATGCATGTGACCTAATGAGGTACCAGCCAGGGTAACAAAGGGACTGCTTGTGTCACTCCCT

TCCCCATCCCAGGCAGCATAGCATGCAGCTCCAGGAGAGACTCCTTCCTTCCACTTGAGGAGAAGAGAAGGGAGAGTAAAGGGGACCTT

TTCTTAGAACTTTGATACCAGCACAGCTGCAGTAGAATAGGGCACCAAGCAGAGCCCTGAGG
```
